# Genetic Variants at Newly Identified Lipid Loci Are Associated with Coronary Heart Disease in a Chinese Han Population

**DOI:** 10.1371/journal.pone.0027481

**Published:** 2011-11-14

**Authors:** Li Zhou, Hu Ding, Xiaomin Zhang, Meian He, Suli Huang, Yujun Xu, Ying Shi, Guanglin Cui, Longxian Cheng, Qing K. Wang, Frank B. Hu, Daowen Wang, Tangchun Wu

**Affiliations:** 1 Institute of Occupational Medicine and the Ministry of Education Key Lab of Environment and Health, School of Public Health, Huazhong University of Science and Technology, Wuhan, Hubei, China; 2 Institute of Hypertension and Department of Internal Medicine, Tongji Hospital, Huazhong University of Science and Technology, Wuhan, Hubei, China; 3 Department of Cardiology, Union Hospital, Huazhong University of Science and Technology, Wuhan, Hubei, China; 4 Key Laboratory of Molecular Biophysics of the Ministry of Education, College of Life Science and Technology and Center for Human Genome Research, Huazhong University of Science and Technology, Wuhan, Hubei, China; 5 Department of Nutrition and Epidemiology, Harvard School of Public Health, Boston, Massachusetts, United States of America; Northwestern University, United States of America

## Abstract

**Background:**

Recent genome-wide association studies (GWAS) have mapped several novel loci influencing blood lipid levels in Caucasians. We sought to explore whether the genetic variants at newly identified lipid-associated loci were associated with CHD susceptibility in a Chinese Han population.

**Methodology/Principal Findings:**

We conducted a two-stage case-control study in a Chinese Han population. The first-stage, consisting of 1,376 CHD cases and 1,376 sex and age- frequency matched controls, examined 5 novel lipid-associated single-nucleotide polymorphisms (SNPs) identified from GWAS among Caucasians in relation to CHD risk in Chinese. We then validated significant SNPs in the second-stage, consisting of 1,269 cases and 2,745 controls. We also tested associations between SNPs within the five novel loci and blood lipid levels in 4,121 controls. We identified two novel SNPs (rs599839 in *CELSR2-PSRC1-SORT1* and rs16996148 in *NCAN-CIL*P2) that were significantly associated with reduced CHD risk in Chinese (odds ratios (95% confidence intervals) in the dominant model 0.76 (0.61-0.90; *P* = 0.001), 0.67 (0.57-0.77; *P* = 3.4×10^−8^), respectively). Multiple linear regression analyses using dominant model showed that rs599839 was significantly associated with decreased LDL levels (*P* = 0.022) and rs16996148 was significantly associated with increased LDL and HDL levels (*P* = 2.9×10^−4^ and 0.001, respectively).

**Conclusions/Significance:**

We identified two novel SNPs (rs599839 and rs16996148) at newly identified lipid-associated loci that were significantly associated with CHD susceptibility in a Chinese Han population.

## Introduction

Coronary heart disease (CHD) is the leading causes of morbidity, mortality and disability in industrialized countries and the prevalence of the disease is increasing rapidly in developing countries [1]–[4]. World Health Organization (WHO) estimates that CHD will be the leading cause of death in the world from 2002 to 2030[2]. Compelling evidence has demonstrated that blood lipid levels including increased levels of total cholesterol (TC), triglycerides (TG), low-density lipoprotein cholesterol (LDL), and decreased levels of high-density lipoprotein cholesterol (HDL) are key modifiable risk factors for CHD [5]–[7]. Family studies and candidate gene studies suggested that genetic variants influencing lipid levels have also been associated with increased susceptibility to CHD [8]–[10].

Genome-wide association studies (GWAS) and targeted gene-based resequencing, have facilitated the gene discovery for complex diseases [11], [12]. Recently, independent GWAS have identified several loci that influence blood lipid concentrations and CHD risk in Caucasian populations [13], [11], [14]–[17]. These loci include the genes such as *APOB*, *APOA5-APOA4-APOC3-APOA1* and *APOE-APOC1-APOC4-APOC2*. Many of these genes have been reported in candidate gene studies over the past 30 years [18], [19]. Recent studies have also identified several novel loci for blood lipid levels and CHD in Caucasians, such as *SORT1* locus and *TRIB1* locus [13]–[15], [17]. However, the associations between these novel genetic variants, lipid levels and risk of CHD are not well established in various ethnic groups.

In this study, we focused on five novel genetic variants associated with lipid levels in previous GWAS. Meta-analysis showed that the five SNPs may have the strongest association with CHD risk in the novel identified lipid-associated loci in Caucasian populations [13], [17]. These variants include the single-nucleotide polymorphisms (SNPs) rs599839 on 1p13.3 (*CELSR2-PSRC1-SORT1* gene), rs17321515 on 8q24 (*TRIB1* gene), rs16996148 on 19p13 (*NCAN-CILP2* gene), rs12695382 on chromosome 3 (*B4GALT4* gene), and rs2254287 on chromosome 6 (*B3GALT4* gene). We conducted a two-stage case-control study to identify the genetic variants at newly identified, lipid-associated loci associated with CHD susceptibility in Chinese. The first-stage, consisting of 1,376 CHD cases and 1,376 sex and age-frequency matched controls, was used to examine 5 novel lipid-associated SNPs in GWAS in Caucasians, and the significant SNPs were validated in the second-stage, consisting of 1,269 cases and 2,745 controls. In addition, we examined the associations between genetic variants and lipid levels in 4,121 control subjects.

## Results

### Characteristics of Study Population

The general characteristics of the two-stage case-control study populations are shown in Table 1. The first case-control study consisted of 1,376 cases and 1,376 controls and the replication study included 1,269 cases and 2,745 controls. TC and LDL levels were significantly lower in cases than in controls, which could be the result of cholesterol-lowering medication in the patients after they were diagnosed. The proportion of taking cholesterol-lowering medicine such as statin in the cases in our study was 67%. The subjects were all of self-reported Chinese Han population.

**Table 1:** General Characteristics of CHD Patients and Controls.

Variable	Stage 1			Stage 2		
	Cases(N = 1376)	Controls(N = 1376)	*P*	Cases(N = 1269)	Controls(N = 2745)	*P*
Sex, m/f, (%)	1000/376 (72.7/27.3)	958/418 (69.6/30.4)	0.08	971/298 (76.5/23.5)	1844/901 (67.2/32.8)	<0.01
Age, mean ± SD	60.1±9.8	60.6±10.0	0.13	60.5±10.4	60.6±8.5	0.73
Body mass index, kg/m^2^	24.5±3.8	23.9±3.5	<0.01	24.4±3.4	24.7±6.0	0.11
Smoking, no/yes, (%)	524/850 (38.1/61.9)	710/666 (51.6/48.4)	<0.01	529/730 (42.0/58.0)	1554/1148 (57.5/42.5)	<0.01
Fasting glucose, mmol/L	6.6±3.5	5.3±2.0	<0.01	6.5±2.4	4.9±1.3	<0.01
Total cholesterol, mmol/L	4.4±1.1	4.7±1.0	<0.01	4.4±1.1	4.6±1.1	<0.01
Triglyceride, mmol/L	1.7±1.2	1.6±1.3	0.81	1.8±1.3	1.6±1.6	<0.01
HDL, mmol/L	1.2±0.4	1.0±0.3	<0.01	1.1±0.4	1.3±0.4	<0.01
LDL, mmol/L	2.6±0.9	2.7±0.8	<0.01	2.5±0.9	2.7±1.0	<0.01

### Association with Lipid Levels

We investigated the association between five novel SNPs previously identified in Caucasians and lipid levels in 4,121 healthy control subjects. SNPs rs599839 and rs16996148 were relatively rare (both MAF<0.10) in our control subjects, which were consistent with the genotyping data for Chinese Han population in the HapMap database. Results of the multiple linear regression analyses adjusted for age, gender, body mass index (BMI) and smoke status are shown in table 2 and Table S1.

**Table 2:** Associations between lipid levels and SNPs in 4121 healthy control subjects in Chinese population.

SNP	Chr	Nearby genes	Genotype	MAF*		*P* Value †			
				Chinese	Caucasians	TC	TG	HDL	LDL
rs599839‡	1	*PSRC1*	A/G	0.073	0.331	0.077	0.811	0.921	0.022
rs16996148‡	19	*NCAN/CILP2*	G/T	0.091	0.095	0.343	0.603	0.001	2.9×10^−4^
rs2254287	6	*B3GALT4*	C/G	0.277	0.356	0.021	0.610	0.790	0.141
rs12695382	3	*B4GALT4*	A/G	0.237	0.129	0.820	0.561	0.344	0.809
rs17321515	8	*TRIB1*	A/G	0.421	0.398	0.535	0.018	0.605	0.961

*MAF the frequency of the minor allele. The data of Chinese was estimated from the genotype data of control subjects in our study.

The data of Caucasians was from HapMap database.

† *P* Value of the lipid levels was calculated by multiple linear regression model adjusted for age, sex, smoking, BMI.

‡ Associations of genotypes (rs599839 and rs16996148) with lipid levels were homozygous minor allele and heterozygous compared with homozygous major allele.

We observed significant associations between rs2254287 and decreased TC levels (*P* = 0.021), rs17321515 and increased TG levels (*P* = 0.018) in additive model. Because of the rarity of the rs599839 and rs16996148 variants in Chinese population, we carried out analyses under additive and dominant models of inheritance. Under an additive model, only rs16996148 showed a statistically significant association with HDL levels (*P* = 0.012). There were borderline associations between rs599839 and LDL levels (*P* = 0.066), rs16996148 and LDL levels (*P* = 0.087). However, when we used dominant model, the associations between SNPs rs599839, rs16996148 and LDL levels became much stronger (*P* = 0.022 and 2.9×10^−4^, respectively).

### Association with Risk of Coronary Heart Disease

We investigated the association between the 5 novel lipid-associated SNPs (rs599839, rs16996148, rs2254287, rs12695382 and rs17321515) previously identified in Caucasians and risk of CHD. Two of these SNPs were significantly associated with CHD in Chinese population. They were rs599839 on 1p13.3 (*CELSR2-PSRC1-SORT1*gene) and rs16996148 on 19p13 (*NCAN-CILP2* gene). As shown in Table 3, in both stages of the case-control analyses, subjects with *AG* genotype of rs599839 and *GT* genotype of rs16996148 were associated with significantly decreased risk of CHD, after adjusting for conventional CHD risk factors such as age, gender, BMI, smoking, blood pressure, glucose levels, and lipid levels (Stage 1, *P* = 0.002 and 0.008; stage 2, *P* = 0.032 and 0.007, respectively). After pooling the data from the two stages, the combined rs599839*AG*+*GG* genotypes and rs16996148*GT*+*TT* genotypes had 0.76- and 0.67- fold lower CHD risk (*P* = 0.001 and 3.4×10^−8^, respectively), compared with the rs599839*AA* and rs16996148*GG* genotypes. There were dose-response effects of rs599839*G* and rs16996148*T* alleles (*P*_trend_ = 4.2×10^−4^ and 1.0×10^−6^, respectively). However, there was no significant association between rs2254287, rs12695382, rs17321515 and CHD risk in Chinese (*P*>0.05) (Table 4).

**Table 3:** Genotype frequencies of the two SNPs in CHD patients and controls and their associations with risk of CHD in Chinese.

Genotype	Stage 1 (1376/1376) *			Stage 2 (1269/2745) *			Pooled (2645/4121) *		
	% †	OR (95% *CI*) ‡	*P*	% †	OR (95% *CI*) ‡	*P*	% †	OR (95% *CI*) ‡	*P*
rs599839									
*AA*	89.7/86.1	1.00(Reference)		88.7/85.9	1.00(Reference)		89.1/86.1	1.00(Reference)	
*AG*	9.7/13.2	0.65(0.47-0.88)	0.002	10.8/13.4	0.77(0.61-0.98)	0.032	10.4/13.2	0.76(0.63-0.91)	0.001
*GG*	0.6/0.7	0.67(0.24-2.08)	0.497	0.5/0.7	0.70(0.25-1.80)	0.481	0.5/0.7	0.68(0.34-1.30)	0.224
*AG+GG*	10.3/13.9	0.74(0.59-0.94)	0.020	11.3/14.1	0.77(0.62-0.97)	0.024	10.9/13.9	0.76(0.61-0.90)	0.001
rs16996148									
*GG*	89.8/85.0	1.00 (Reference)		85.3/81.3	1.00(Reference)		87.6/82.6	1.00(Reference)	
*GT*	9.6/14.2	0.66(0.51-0.91)	0.008	14.7/17.8	0.78(0.63-0.94)	0.007	12.1/16.5	0.68(0.57-0.79)	5.0×10^−7^
*TT*	0.6/0.8	1.02(0.35-2.92)	0.988	0.0/0.9	-	0.998	0.3/0.9	0.26(0.13-0.62)	0.002
*GT + TT*	10.2/15.0	0.67(0.50-0.86)	0.001	14.7/18.7	0.73(0.61-0.87)	0.001	12.5/17.4	0.67(0.57-0.77)	3.4×10^−8^

*Number of cases/number of controls.

† % of cases/% of controls.

‡ Data were calculated by logistic regression analysis with adjustment for age, sex, smoking, BMI, blood pressure, glucose levels, and lipid levels.

**Table 4:** Genotype frequencies of the three SNPs in CHD patients and controls and their associations with risk of CHD in Chinese.

Genotype	Stage 1 (1376/1376) *		
	% †	OR (95% *CI*) ‡	*P*
rs2254287			
*GG*	9.0/8.6	1.00 (Reference)	
*GC*	38.5/38.3	1.05(0.74–1.47)	0.832
*CC*	52.5/53.1	0.99(0.70–1.38)	0.939
rs12695382			
*GG*	5.8/4.8	1.00 (Reference)	
*GA*	36.7/37.9	0.79(0.55–1.26)	0.225
*AA*	57.5/57.3	0.77(0.52–1.15)	0.190
rs17321515			
*AA*	19.9/17.9	1.00 (Reference)	
*AG*	47.9/48.4	0.94(0.70–1.18)	0.467
*GG*	32.2/33.7	0.88(0.63–1.10)	0.235

*Number of cases/number of controls.

† % of cases/% of controls.

‡ Data were calculated by logistic regression analysis with adjustment for age, sex, smoking, BMI, blood pressure, glucose levels/Diabetes status, and lipid levels.

We conducted stratified analyses for SNPs rs599839 and rs16996148 (Table S2). Stratification analyses using pooled case-control sets showed that the risk associated with the rs599839 was significantly different between the subgroups by gender and smoke status. rs599839*AG*+*GG* genotypes were associated with lower risk in males (OR = 0.71, 95% CI 0.59 to 0.86) and smokers (OR = 0.68, 95% CI 0.55 to 0.85). The associations between the two SNPs and CHD risk were all significant in other subgroups under a dominant model. Although the associations between the two SNPs and lipid levels were found in control subjects, we stratified the case-control sets by means of the lipid levels in our study. There were significant associations except the rs599839 and higher TG levels subgroup. Furthermore, when multiplicative interaction was tested for each possible pair of these two SNPs, we found significant interaction between rs16996148 and HDL levels (*P* = 0.006).

## Discussion

In the present study, we examined whether the genetic variants at the newly identified, lipid-associated loci in Caucasians [13]–[15], [17] are also associated with CHD susceptibility in Chinese population. We identified two SNPs (rs599839 and rs16996148) that were significantly associated with CHD risk in two large independent case-control studies. Furthermore, we also provided evidence for four SNPs (rs599839, rs16996148, rs2254287 and rs17321515) that influence lipid levels in Chinese.

The SNP rs599839 in *CELSR2-PSRC1-SORT1* on 1p13 showed strong evidence for associations with LDL levels and risk of CHD in our study, which was consistent with the results from previous studies in Caucasians [20], [21], [16]. Sandhu et al using GWAS data from 11,685 European participants firstly identified rs599839 was associated with LDL levels^17^. Willer et al reported that rs599839 was associated with both LDL levels and CHD risk in Caucasians^15^. One possibility is that rs599839 or an associated variant influences expression of *SORT1*, a nearby gene that mediates endocytosis and degradation of lipoprotein lipase. Musunuru et al. have provided functional evidence for this novel regulatory pathway for lipoprotein metabolism and suggested that modulation of this pathway might alter risk for myocardial infarction [22].

Willer et al reported that rs16996148 on chromosome 19p13 near *NCAN-CILP2* gene region was associated with LDL, TG levels and CHD risk in Europeans [17]. Tai et al recently reported that rs16996148 was significantly associated with HDL levels in an Asian Malay population [23]. In the present study, rs16996148 was associated with LDL, HDL levels, but not associated with TG levels in Chinese population. We found significant associations of minor allele *T* with higher LDL and HDL levels. In addition, the presence of the minor allele *T* was also significantly associated with a decreased risk of CHD in our study. These differences might be due to heterogeneity among the different populations or the differential impact of comparable differences in these lipids on risk of CHD. It suggested that rs16996148 may influence risk of CHD through an effect on the HDL levels in Chinese subjects. Alternatively, this genetic variant may have a direct, non-LDL-mediated effect on the risk of CHD. The roles that *NCAN* or *CILP2* might play in lipoprotein metabolism are unclear until now. Fine-mapping and functional studies, including large-scale resequencing might help identify these novel and rare functional genetic variants in the future.

As previously shown in Europeans, rs17321515 adjacent to *TRIB1* was significantly associated with TG levels in Chinese population. This is the first report that rs2254287 was significantly associated with TC levels in Chinese population. Willer et al reported that rs17321515 and rs2254287 were significantly associated with LDL and risk of CHD in Europeans [17]. Tai et al recently reported that rs17321515 was significantly associated with increased cardiovascular disease in an Asian Malay population [23]. However, it should be noted that the age and gender were not matched in the cases and controls in Malay study. On the other hand, there were only 327 cardiovascular disease cases and all cases were self-reported in their study, which might lead to some selection bias.

Lipid measurements may be affected by dietary and treatment factors as well as laboratory errors. By contrast, genotypes are invariant over time and can be precisely measured on a single assay, and thus can exert an effect on lipid levels over a lifetime. Furthermore, the newly identified loci may lead to important new insights about lipid metabolism and atherogenesis mechanism. Potential new lipid-associated loci could contribute to prevention strategies. It also provides evidence for the development of novel therapeutics, and influences optimal treatment profiles for each individual, and then results in improved management of blood lipid levels and reduction of CHD risk.

The present study has several strengths. First, the two-population based studies provided sufficient statistical power for testing the association between rs599839, rs16996148 and CHD risk and reduced the false positive report probability. Second, the subjects used to test the association between the genotype and phenotype (lipid levels) were healthy individuals who were not receiving lipid-lowering medications or with elevated blood lipids. However, because the studied SNPs are uncommon in Chinese populations, even larger samples are needed to confirm our findings. In addition, large-scale prospective studies are warranted to validate the values of genetic variants and biomarkers in predicting subsequent risk of CHD.

In conclusion, we have identified two novel SNPs at newly identified, lipid-associated loci that are significantly associated with CHD susceptibility in a Chinese Han population. Functional analyses are warranted to elucidate the biological plausibility of the novel identified genetic variants in the development of atherosclerosis and CHD.

## Materials and Methods

### Ethics Statement

The Ethics Committee of Tongji Medical College approved the present study, and written informed consent was obtained from all subjects.

### Study Population

We performed two independent case–control analyses. The first stage consisted of 1,376 patients with CHD and 1,376 age- and sex-frequency matched healthy control subjects. The study design has been described elsewhere [24]. The patients were consecutively recruited from 3 hospitals (Tongji Hospital, Union Hospital, and Wugang Hospital) in Wuhan (Hubei, China) between May 2004 and October 2006. The second stage, which was used to confirm the results obtained from the first stage, consisted of 1,269 CHD cases and 2,745 controls derived from the Tongji Hospital in Wuhan (Hubei, China) between May 2007 and October 2009. The inclusion criteria for the case subjects were stenoses ≥ 50% in at least 1 major coronary artery by coronary angiography and/or a diagnosis of CHD based on the World Health Organization criteria [25]. The control subjects in two stages, residing in the same communities as the cases, were determined to be free of CHD and peripheral atherosclerotic arterial disease by medical history, clinical examinations, and electrocardiography. Study subjects who were receiving lipid-lowering medication or dyslipidemia were excluded from the controls.

Structured questionnaires were used by trained interviewers to collect information on demographic variables, medical history, medications, and lifestyle habits (including smoking). Subjects were classified as smokers and nonsmokers. Those who had smoked less than 100 cigarettes in the lifetime were defined as nonsmokers; otherwise, they were defined as smokers. Body mass index (BMI) was calculated as weight in kilograms divided by the square of height in meters.

### Determination of Blood Lipid Levels

Blood samples were collected after an overnight (at least 12 h) fasting. TC, TG, HDL and LDL levels in blood were measured according to standard enzymatic methods on a MINDRAY BS-200 analyser (MINDRAY, Shenzhen, China).

### Genotyping

Fasting venous blood was collected in 5-ml EDTA tubes, and genomic DNA was isolated with a Puregene kit (Gentra Systems, Inc., Minneapolis, MN, USA). Genotyping was performed with TaqMan SNP allelic discrimination by means of an ABI 7900HT (Applied Biosystems, Foster City, CA, USA). A successful genotyping rate of over 98% was achieved for the entire SNPs test. About 5% of the samples were repeated and the concordance was 100%. All SNPs were in Hardy-Weinberg equilibrium (*P*>0.05).

### Statistical Analyses

Continuous variables were reported as the mean value ± SD. Normal distribution of data was analyzed using the Kolmogorov-Smirnov normality test. Data with a normal distribution were compared by Student's t-test, and those with unequal variance or without a normal distribution were analyzed by a Mann-Whitney rank sum test. Categorical values were compared by the chi-square test, which was also used to test for deviation of genotype distribution from Hardy-Weinberg equilibrium. The association between SNPs and CHD risk was estimated by computing odds ratios (ORs) and 95% confidence intervals (CIs) from the multivariate logistic regression analyses. The effects of genotypes on plasma lipid levels were assessed by multiple linear regression models. The probability level accepted for significance was *P*<0.05. The significance of multiplicative interactions between the selected SNPs and covariates was determined by the likelihood ratio test using the logistic regression model. All data analyses were carried out with the statistical analysis software package SPSS 16.0 (SPSS Inc., Chicago, IL, USA).

## Supporting Information

Table S1 Associations between SNPs at newly identified lipid-associated loci with lipid levels in Chinese.
(DOC)

Table S2 Stratification analysis for association between two SNPs genotypes and risk of CHD.
(DOC)
