# Supplementary material for: Genetic Variants at Newly Identified Lipid Loci Are Associated with Coronary Heart Disease in a Chinese Han Population
Source: PLoS One. 2011 Nov 14;6(11):e27481. doi: 10.1371/journal.pone.0027481 (PMC3215720; doi:10.1371/journal.pone.0027481)
Supplement: Table S1 — Associations between SNPs at newly identified lipid-associated loci with lipid levels in Chinese. (DOC) [file pone.0027481.s001.doc]

**Table S1. Associations between SNPs at newly identified lipid-associated loci with lipid levels in Chinese.**

| **Variable** | **Genotypes**^*^ | | | | ***P*** † |
| --- | --- | --- | --- | --- | --- |
|  | ***Hom Min*** | | ***Het*** | ***Hom Maj***^*^ |  |
| **TC (mmol/l)** |  | |  |  |  |
| rs599839 | 4.80 ± 0.20 | | 4.60 ± 0.05 | 4.70 ± 0.02 | 0.629 |
|  | 4.61 ± 0.05 | | | 4.70 ± 0.02 | 0.077 |
| rs16996148 | 4.88 ± 0.18 | 4.71 ± 0.04 | | 4.67 ± 0.02 | 0.261 |
|  | 4.72 ± 0.04 | | | 4.67 ± 0.02 | 0.343 |
| rs2254287 | 4.55 ± 0.10 | | 4.77 ± 0.05 | 4.80 ± 0.04 | 0.021 |
| rs12695382 | 4.89 ± 0.09 | | 4.92 ± 0.03 | 4.91 ± 0.03 | 0.820 |
| rs17321515 | 4.89 ± 0.04 | | 4.92 ± 0.03 | 4.93 ± 0.05 | 0.535 |
| **TG (mmol/l)** |  | |  |  |  |
| rs599839 | 1.67 ± 0.23 | | 1.59 ± 0.05 | 1.61 ± 0.02 | 0.772 |
|  | 1.59 ± 0.05 | | | 1.61 ± 0.02 | 0.811 |
| rs16996148 | 1.62 ± 0.20 | 1.58 ± 0.05 | | 1.61 ± 0.02 | 0.941 |
|  | 1.58 ± 0.04 | | | 1.61 ± 0.02 | 0.603 |
| rs2254287 | 1.64 ± 0.13 | | 1.64 ± 0.06 | 1.71 ± 0.05 | 0.610 |
| rs12695382 | 1.47 ± 0.11 | | 1.53 ± 0.04 | 1.53 ± 0.03 | 0.561 |
| rs17321515 | 1.62 ± 0.05 | | 1.54 ± 0.03 | 1.48 ± 0.04 | 0.018 |
| **HDL (mmol/l)** |  | |  |  |  |
| rs599839 | 1.18 ± 0.08 | | 1.21 ± 0.02 | 1.21 ± 0.01 | 0.753 |
|  | 1.21 ± 0.02 | | 1.21 ± 0.01 | 0.921 |  |
| rs16996148 | 1.38 ± 0.07 | | 1.25 ± 0.02 | 1.20 ± 0.01 | 0.012 |
|  | 1.26 ± 0.02 | | 1.20 ± 0.01 | 0.001 |  |
| rs2254287 | 1.03 ± 0.03 | | 1.04 ± 0.02 | 1.02 ± 0.01 | 0.790 |
| rs12695382 | 1.29 ± 0.04 | | 1.26 ± 0.02 | 1.25 ± 0.01 | 0.344 |
| rs17321515 | 1.26 ± 0.02 | | 1.25 ± 0.01 | 1.24 ± 0.02 | 0.605 |
| **LDL (mmol/l)** |  | |  |  |  |
| rs599839 | 2.55 ± 0.02 | | 2.63 ± 0.04 | 2.72 ± 0.02 | 0.066 |
|  | 2.63 ± 0.04 | | 2.72 ± 0.02 | 0.022 |  |
| rs16996148 | 2.94 ± 0.15 | | 2.81 ± 0.04 | 2.68 ± 0.02 | 0.087 |
|  | 2.82 ± 0.03 | | 2.68 ± 0.02 | 2.9×10^-4^ |  |
| rs2254287 | 2.61 ± 0.08 | | 2.69 ± 0.04 | 2.74 ± 0.03 | 0.141 |
| rs12695382 | 2.87 ± 0.08 | | 2.87 ± 0.03 | 2.85 ± 0.02 | 0.809 |
| rs17321515 | 2.86 ± 0.03 | | 2.85 ± 0.02 | 2.86 ± 0.04 | 0.961 |

**Hom Min* homozygote for the minor allele, *Het* heterozygotes, *Hom Maj* homozygote for the major allele.

† Adjusted for age, sex, smoking, BMI, blood pressure, glucose levels/Diabetes status, and lipid levels.
