# Supplementary material for: Genetic Variants at Newly Identified Lipid Loci Are Associated with Coronary Heart Disease in a Chinese Han Population
Source: PLoS One. 2011 Nov 14;6(11):e27481. doi: 10.1371/journal.pone.0027481 (PMC3215720; doi:10.1371/journal.pone.0027481)
Supplement: Table S2 — Stratification analysis for association between two SNPs genotypes and risk of CHD. (DOC) [file pone.0027481.s002.doc]

**Table S2. Stratification analysis for association between two SNPs genotypes and risk of CHD.**

|  |  | **rs599839 OR (95% CI )**^*^ | | |  | **rs16996148 OR (95% CI )**^*^ | | |
| --- | --- | --- | --- | --- | --- | --- | --- | --- |
|  |  | ***AA*** | ***AG+GG*** | ***P* _interaction_** |  | ***GG*** | ***GT + TT*** | ***P* _interaction_** |
| **Gender** | **Male** | 1.00 | 0.71(0.59-0.86) | 0.248 |  | 1.00 | 0.61(0.51-0.74) | 0.303 |
|  | **Female** | 1.00 | 0.89(0.66-1.19) |  |  | 1.00 | 0.60(0.45-0.81) |  |
| **Age, years** | **≤60** | 1.00 | 0.79(0.63-0.98) | 0.720 |  | 1.00 | 0.54(0.43-0.68) | 0.610 |
|  | **>60** | 1.00 | 0.75(0.60-0.94) |  |  | 1.00 | 0.66(0.53-0.83) |  |
| **Smoking status** | **Smokers** | 1.00 | 0.68(0.55-0.85) | 0.073 |  | 1.00 | 0.59(0.48-0.74) | 0.472 |
|  | **Non-smokers** | 1.00 | 0.87(0.69-1.09) |  |  | 1.00 | 0.63(0.49-0.80) |  |
| **BMI, kg/m^2^** | **<25** | 1.00 | 0.80(0.65-0.98) | 0.929 |  | 1.00 | 0.63(0.51-0.78) | 0.348 |
|  | **≥25** | 1.00 | 0.74(0.58-0.94) |  |  | 1.00 | 0.57(0.44-0.74) |  |
| **LDL, mmol/l** | **< 2.6** | 1.00 | 0.67(0.53-0.88) | 0.954 |  | 1.00 | 0.64(0.50-0.85) | 0.057 |
|  | **≥ 2.6** | 1.00 | 0.76(0.59-0.97) |  |  | 1.00 | 0.52(0.43-0.67) |  |
| **TC, mmol/l** | **< 4.5** | 1.00 | 0.75(0.62-0.94) | 0.915 |  | 1.00 | 0.71(0.57-0.85) | 0.657 |
|  | **≥ 4.5** | 1.00 | 0.72(0.55-0.91) |  |  | 1.00 | 0.56(0.46-0.75) |  |
| **TG, mmol/l** | **< 1.7** | 1.00 | 0.72(0.59-0.88) | 0.850 |  | 1.00 | 0.68(0.57-0.82) | 0.228 |
|  | **≥ 1.7** | 1.00 | 0.84(0.64-1.11) |  |  | 1.00 | 0.59(0.45-0.79) |  |
| **HDL, mmol/l** | **< 1.15** | 1.00 | 0.79(0.63-0.98) | 0.404 |  | 1.00 | 0.75(0.60-0.92) | 0.006 |
|  | **≥ 1.15** | 1.00 | 0.71(0.55-0.90) |  |  | 1.00 | 0.56(0.44-0.70) |  |

^*^ ORs were obtained from a logistic regression model with adjustment for age, sex, smoking, BMI and lipid-lowering medication use.
